# Severe Pneumonia Due to Streptococcal Toxic Shock Syndrome in a Patient Positive for Influenza Virus Antigen: A Case Report

**DOI:** 10.7759/cureus.83620

**Published:** 2025-05-06

**Authors:** Kazushi Nagai, Ryota Inokuchi, Hiroyuki Nakano, Toshifumi Asada, Ryohei Horie, Tomoki Wada, Miyuki Yamamoto, Kent Doi

**Affiliations:** 1 Emergency and Critical Care Medicine, The University of Tokyo Hospital, Bunkyo-ku, JPN

**Keywords:** coinfections, group a streptococcus, influenza virus, pneumonia, streptococcal toxic shock

## Abstract

Since the onset of the COVID-19 pandemic, the incidence of streptococcal toxic shock syndrome (STSS) has increased. Additionally, complications involving influenza virus infections in STSS cases have been reported more frequently than in the pre-pandemic period. We report the case of a 69-year-old man who presented to the ED with progressive dyspnea and fever, without any signs suggestive of soft tissue infection. Chest CT revealed right-sided pneumonia, and a rapid influenza test returned positive. On the second day of hospitalization, *Streptococcus pyogenes* (emm1) was isolated from both blood and sputum cultures. Despite aggressive intensive care, including fluid resuscitation, vasoactive support, mechanical ventilation, and renal replacement therapy, the patient’s condition deteriorated rapidly. He died 24 hours after admission to the ICU. STSS is a rapidly progressing disease with a high mortality rate, capable of causing severe clinical decline within a short time. Clinicians should remain vigilant for the rising occurrence of STSS and influenza coinfections during the COVID-19 era to improve differential diagnosis and optimize early treatment strategies.

## Introduction

Streptococcal toxic shock syndrome (STSS) is a potentially fatal condition that can cause multiple organ failure within 24 hours [1]. It is primarily triggered by infection with Group A *Streptococcus *(GAS) and typically presents as a skin and soft tissue infection. However, STSS can also manifest as a respiratory tract infection or pelvic inflammatory disease in some patients [2]. Since the onset of the COVID-19 pandemic, the global incidence of STSS has been on the rise [3]. In Japan, the number of reported STSS cases is expected to reach a record high in 2023 [4]. Coinfection with GAS and seasonal influenza may result in rapidly progressive pneumonia and systemic complications, underscoring the clinical significance of such cases. Here, we report a case of STSS in a patient who tested positive on a rapid influenza antigen test and developed severe pneumonia due to GAS infection.

## Case presentation

A man in his late 60s, with a history of hypopharyngeal cancer and a permanent tracheostoma but no other significant medical history, was admitted to the ED with respiratory distress. When evaluated by the paramedic, his oxygen saturation (SpO₂) was 72% on room air. Upon arrival at the hospital, the patient exhibited a mild consciousness disorder. His vital signs were as follows: blood pressure 100/60 mm Hg, pulse rate 130 beats per minute, body temperature 38.2°C, respiratory rate 32 breaths per minute, SpO₂ 82% on a reservoir oxygen mask at 10 liters per minute, and a Glasgow Coma Scale score of E3VtM5. A tracheostomy tube was promptly inserted through the permanent tracheostoma, and manual ventilation was initiated, which improved his oxygenation to 100%. An arterial blood gas analysis revealed metabolic acidosis, hyperlactatemia, and hypoglycemia (Table 1). After the administration of 20 g of IV glucose, his consciousness disorder improved.

**Table 1:** Arterial blood gas values at admission in a patient receiving 10 l/min of oxygen via a reservoir mask AG, anion gap; Hb, hemoglobin

Arterial blood gas (normal range)	Result
pH (7.35-7.45)	7.312
pCO₂ (35.0-45.0 mmHg)	29.1 mmHg
pO₂ (83.0-108.0 mmHg)	147 mmHg
HCO₃⁻ (22.5-26.9 mmol/L)	14 mmol/L
Hb (13.5-17.5 g/dL)	14.3 g/dL
AG (12-16 mmol/L)	20.5 mmol/L
Na (135-148 mmol/L)	136 mmol/L
K (3.5-4.5 mmol/L)	4.9 mmol/L
Cl (98-108 mmol/L)	107 mmol/L
Glu (73-109 mg/dL)	47 mg/dL
Lac (0.5-2.0 mmol/L)	6.4 mmol/L

Investigations

Blood examinations revealed reduced white blood cell and platelet counts, elevated liver enzyme levels, decreased renal function, and elevated brain natriuretic peptide (Table 2). The patient scored six on the JAAM-DIC diagnostic criteria. This scoring system, introduced by the Japanese Association for Acute Medicine in 2006, helps facilitate early diagnosis and therapeutic intervention for disseminated intravascular coagulation (DIC). It ranges from 0 to 8 points based on systemic inflammatory response syndrome, platelet count, prothrombin time ratio, and fibrin/fibrinogen degradation products, with a score of 4 or higher indicating DIC.

**Table 2:** Laboratory findings on days 1 and 2 of hospital admission APTT, activated partial thromboplastin time; AST, aspartate aminotransferase; ALT, alanine aminotransferase; BNP, B-type natriuretic peptide; BUN, blood urea nitrogen; CK, creatine kinase; Hb, hemoglobin; hsc TnI, high-sensitivity troponin I; Plt, platelets; PT, prothrombin time; T-Bil, total bilirubin; γ-GTP, gamma-glutamyl transferase

Laboratory data (normal range)	Day 1 (ER)	Day 2 (EICU)
WBC (3,300-8,600 /μL)	1,800	5,700
Hb (13.7-16.8 g/dL)	13.9	12.9
Plt (158-348 × 10³/μL)	14.6	9
Na (138-145 mmol/L)	138	137
K (3.6-4.8 mmol/L)	5.3	5.7
BUN (8-20 mg/dL)	57.4	67.8
Cre (0.65-1.07 mg/dL)	2.9	4.13
AST (13-30 U/L)	3,935	3,656
ALT (10-42 U/L)	1,695	1,205
γ-GTP (13-64 U/L)	90	64
T-Bil (0.4-1.5 mg/dL)	2	2.1
CK (59-248 U/L)	23,368	33,344
Mb (17.4-105.7 ng/mL)	48,013	47,920
PT (86.0-124.1%)	20.8	<15.0
APTT (24.0-34.0 seconds)	36.5	72
D-dimer (<1.0 μg/mL)	49.8	49.4
hsc TnI (<26.2 ng/L)	944.9	1,156.40
CRP (<0.30 mg/dL)	6.9	12.5
BNP (<18.4 pg/mL)	>5,000	

**Figure 1:**
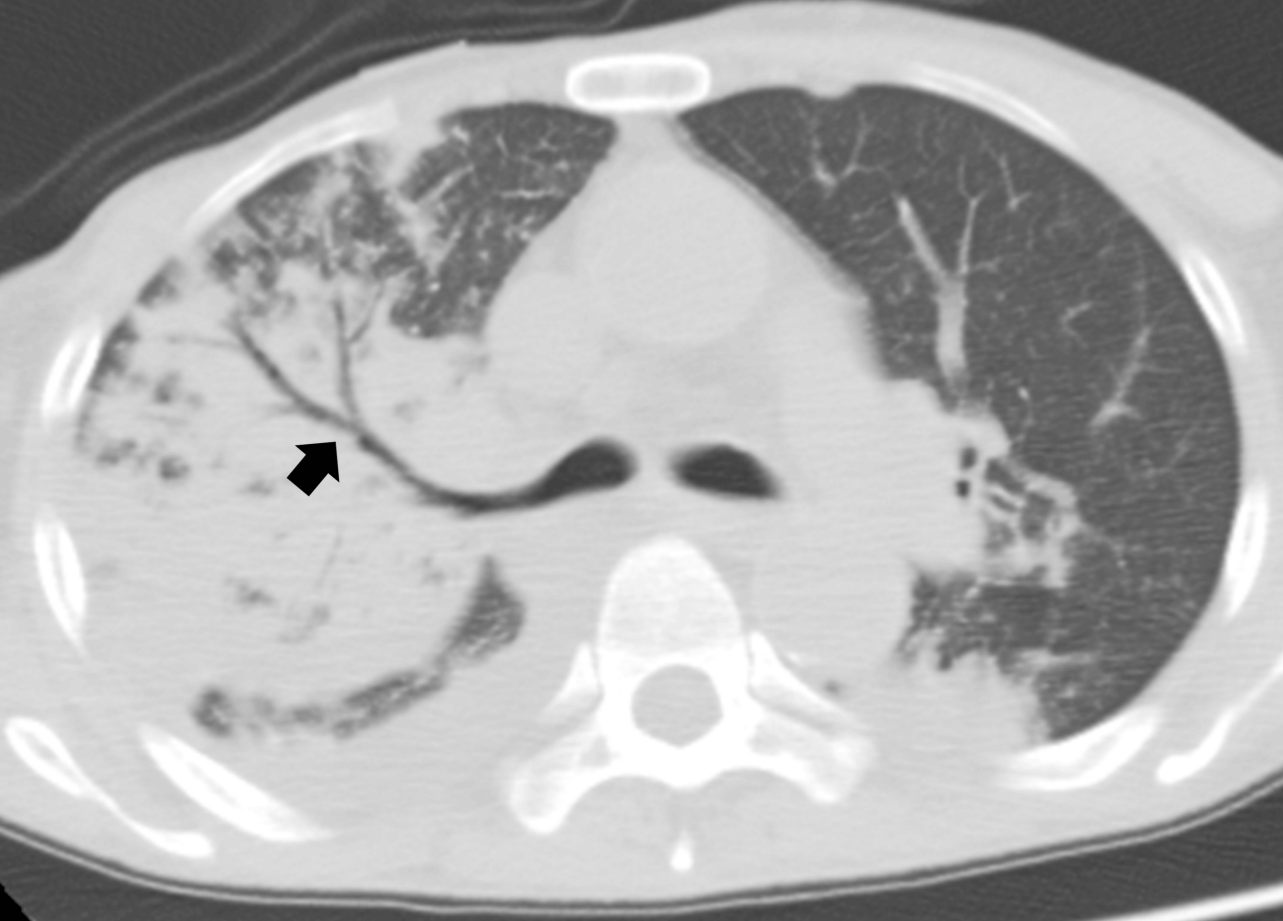
Chest CT scan Chest CT revealed an extensive infiltrative shadow with an air bronchogram (arrow) in the right lung.

Cardiac echocardiography revealed reduced left ventricular contractility. Chest CT showed an extensive infiltrative shadow with an air bronchogram in the right upper lung (Figure 1). The rapid influenza antigen test returned positive results. After collecting blood and sputum samples, the patient was treated with piperacillin/tazobactam (2.25 g every six hours) and peramivir (200 mg).

Treatment

The patient was transferred to the ICU, where he developed anuria, severe metabolic acidosis, and hyperkalemia. Despite the administration of large doses of catecholamines, steroids for relative adrenal failure, and continuous renal replacement therapy, the patient’s condition continued to deteriorate. Blood tests conducted the day after admission revealed a progressive inflammatory response and a coagulation disorder (Table 2).

Outcome and follow-up

On the second day of admission, *Streptococcus pyogenes* was detected in both blood and sputum cultures, confirming the diagnosis of concurrent STSS and influenza viral infection. Genetic testing of the isolated *S. pyogenes *identified the emm1 genotype. Antibiotics were switched from piperacillin/tazobactam to meropenem, vancomycin, and azithromycin. However, the patient’s clinical condition did not improve, and he died 24 hours after ICU admission. The *S. pyogenes *strain later showed good sensitivity to several antibiotics, with minimum inhibitory concentrations of ampicillin ≤0.25, ceftriaxone ≤0.25, and penicillin G ≤0.063.

## Discussion

We present a case of STSS associated with influenza viral infection in a patient without traditional risk factors, such as diabetes, immunosuppression, or skin and soft tissue infection. A strength of this case is our ability to promptly identify the progression of multi-organ failure and initiate intensive care interventions, including mechanical ventilation, vasopressor support, and renal replacement therapy. However, there were several limitations. First, no autopsy was performed, limiting the pathological confirmation of disease processes. Second, antimicrobial susceptibility testing results were obtained only after the initiation of empirical therapy, which may have influenced early treatment decisions. Third, although the severity of the clinical course met the criteria for STSS, IV immunoglobulin (IVIG) therapy, a potential intervention with benefits in STSS, was not administered. Several studies, including systematic reviews, have reported improved outcomes in STSS patients treated with IVIG, particularly when used in combination with clindamycin [5].

An increase in STSS cases during and after the COVID-19 pandemic has been reported in several countries, including Japan [4]. Epidemiological data suggest a potential association with the emergence of the M1UK variant of *S. pyogenes*, characterized by upregulated superantigen production and increased transmissibility [6]. Although detailed genetic testing was not performed in this case, the isolated emm1 genotype has been linked to severe invasive infections. Globally, epidemiological surveillance has shown significant diversity in emm types, with emm1 being one of the most common genotypes associated with invasive disease [7]. Additionally, coinfection with the influenza virus may facilitate the translocation of *S. pyogenes* across respiratory epithelial barriers, contributing to fulminant pneumonia and STSS [8,9]. A recent multicenter study in France found that patients admitted to ICUs with invasive GAS infections were more likely to have concurrent influenza infection during the post-pandemic period [10]. In line with these findings, our case underscores the growing clinical concern regarding the combined effect of GAS and influenza in causing life-threatening infections.

This patient met the diagnostic criteria for STSS, including isolation of *S. pyogenes* from sterile sites, hypotension, and multiple organ involvement. Despite aggressive treatment, his condition deteriorated rapidly, consistent with the fulminant nature of STSS. The presence of influenza coinfection may have exacerbated the inflammatory response and hastened disease progression. Although the delay in obtaining susceptibility results, the absence of IVIG therapy, and the lack of autopsy pose challenges in drawing firm conclusions, the clinical and microbiological findings strongly support the diagnosis and highlight the lessons learned from this case.

STSS should be considered in patients presenting with rapidly progressive pneumonia and a positive influenza antigen test, even in the absence of classic risk factors or soft tissue involvement. Early recognition and prompt initiation of intensive care, along with consideration of adjunctive therapies such as IVIG, may be essential to improve outcomes in similar clinical scenarios.

## Conclusions

Clinicians should consider STSS due to GAS in patients with positive rapid influenza antigen test results and rapidly progressive pneumonia. This case emphasizes that STSS can develop rapidly, even in patients without typical risk factors, and requires immediate recognition and intervention.
